# Know your enemy: understanding mosquito biology to advance malaria elimination in Africa

**DOI:** 10.1007/s00436-025-08534-9

**Published:** 2025-08-18

**Authors:** Kennedy Zembere, Patrick Ken Kalonde, Fanuel Meckson Bickton

**Affiliations:** 1 Malawi-Liverpool-Wellcome Programme, Blantyre, Malawi; 2 Liverpool School of Tropical Medicine, Liverpool, UK; 3 Kamuzu University of Health Sciences, Blantyre, Malawi

**Keywords:** Mosquito biology, *Anopheles* mosquitoes, Vector control, Community engagement, Health education, Sustainable interventions

## Abstract

Over a century has passed since the discovery that the female anopheline mosquito is a vector of malaria, prompting extensive efforts to combat the disease. Vector control remains the primary malaria prevention strategy, accounting for a remarkable 65% reduction in malaria cases between 2000 and 2015. A thorough understanding of the fundamental biology of malaria vectors is essential for national malaria control programmes, governments, and local communities to implement sustainable and effective vector control measures. This article explores the key aspects of mosquito biology, such as adult and juvenile life stages, the intrinsic incubation period, and blood-seeking behaviour, within the context of the mosquito life cycle. By emphasizing the importance of this knowledge, we illustrate how it can inform community-level control initiatives and guide national programmes in designing more effective interventions for vector-borne disease management. Furthermore, the article underscores how researchers can harness these biological insights to develop and deploy innovative tools that have the potential to revolutionize malaria control efforts.

**Graphical Abstract:**

Illustration of how the mosquito lifecycle could be exploited to control mosquito populations by targeting mosquito developmental stages to support effective malaria control initiatives.

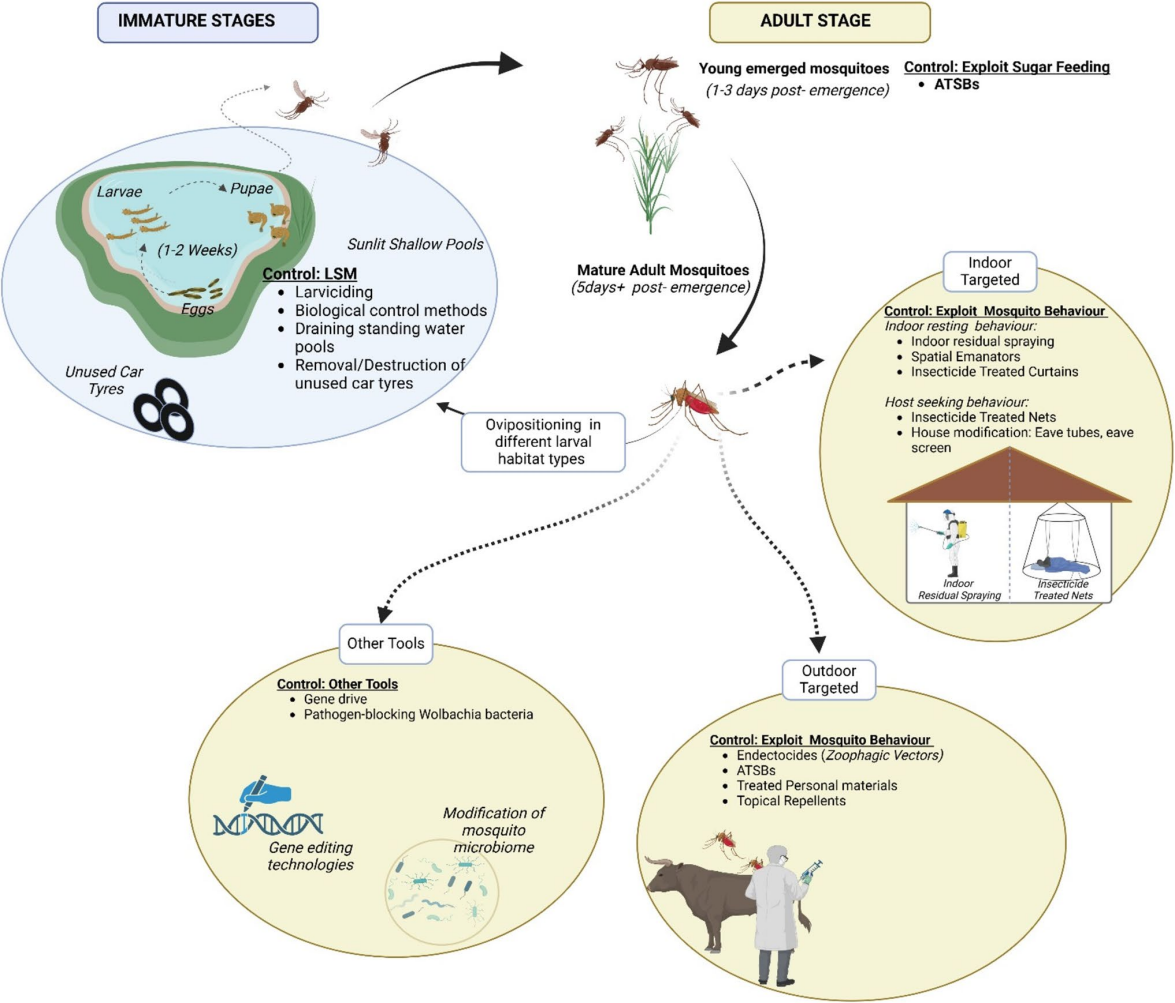

## Introduction

On 20 August 1897, one of the renowned scientists, Sir Ronald Ross, while working on dissected guts of *Anopheles* mosquitoes, discovered that the female *Anopheles* mosquito was responsible for the transmission of malaria, for which he received a Nobel prize in 1902 (CDC 2015). Since that discovery, the world commemorates 20th August as the World Mosquito Day with an aim of raising awareness about mosquitoes and the efforts being put in place to help curb and fight their impact on human health.

Despite control efforts in place, the mosquito remains the world’s deadliest animal (CDC 2019). There are different kinds of mosquitoes, causing various diseases including the deadly malaria. Malaria is transmitted to humans when an infected female *Anopheles* mosquito bites a healthy individual (WHO 2023). In 2021, there were approximately 247 million malaria cases and 593,000 deaths, with over 90% of these cases and deaths coming from Africa (WHO 2024).

The adage, “prevention is better than cure”, aptly applies to malaria control strategies. Vector control, which is a preventive measure aimed at managing or eradicating disease-spreading insects, is regarded as the primary method of preventing malaria and other mosquito-transmitted diseases. According to a study by Bhatt et al. (2015), vector control has contributed significantly to malaria prevention, averting over 65% of all malaria cases between 2000 and 2015 (Bhatt et al. 2015). Additionally, although traditional vector control strategies (insecticide-treated bed nets and indoor residual spraying) have been effective, they do not fully account for the diversity in mosquito ecology and behaviour (Ferguson et al. 2010a; Debrah et al. 2021; Msugupakulya et al. 2023). Changes in ecological characteristics over time may alter habitat suitability of other vectors, increasing their prevalence while reducing the suitability of other malaria vectors (Msugupakulya et al. 2023).

When planning and implementing vector control strategies for sustained malaria control, a good understanding of the biology, ecology, and behaviour of the *Anopheles* mosquitoes is critical (Godfray 2013). This article outlines fundamental facts and behavioural characteristics about the vectors of malaria, often referred to as the “deadliest animals in the world”. We also aim to critically assess how gaps in our understanding of mosquito ecological diversity and how behavioural diversity may hinder malaria control efforts.

By highlighting the role of community health education, this article underscores how equipping local communities (in malaria endemic regions) with basic knowledge about the malaria vector biology and ecology can empower them to adopt cost-effective prevention measures. It further explores how leveraging this knowledge can help malaria control programmes, governments, and researchers to enhance their strategies to combat these lethal and debilitating creatures. To the best of our knowledge, this paper is the first to consolidate evidence on mosquito characteristics and facts and demonstrate their significance in enhancing malaria control efforts, with a particular focus on integrating health education and innovations. The article highlights how basic knowledge of the mosquito biology can enable local communities, malaria control programmes, and governments in Africa to implement more cost-effective and sustainable malaria prevention approaches.

## Notable mosquito facts and how they may be exploited for control

### A mosquito’s juvenile stage takes about 1 to 2 weeks to develop in water

The lifecycle of mosquitoes begins in water when an adult female lays fertilized eggs following a successful mating (Graphical abstract). Eggs typically hatch within 48 h, releasing larvae that are very active and feed voraciously on microorganisms and organic matter. The larvae develop through four stages (first instar, second instar, third instar, and fourth instar larva stages) before molting to pupae (which is less active and non-feeding) after several days before emerging as adult mosquitoes (Abd 2020; Abed and Kareem 2022). Juvenile-stage development in mosquitoes typically spans 1 to 2 weeks from egg to adult, depending on environmental factors such as food availability (Abed and Kareem 2022).

#### Larval source management to target juvenile stages

While the aquatic environment provides the life necessities for the juvenile stages to complete development (Hawkes and Richard 2021), their confinement to one specific location makes them particularly vulnerable to targeted interventions (Tusting et al. 2013). This is because human interventions targeting their habitats can significantly disrupt their development, rendering the juvenile stages helpless (Tusting et al. 2013). Anthropogenic activities such as larval source management (LSM), that can disturb or manage these aquatic habitats, can effectively disrupt the mosquito life cycle and reduce mosquito populations that survive to adulthood (WHO 2013).

Larval source management (LSM) is one historical approach that has been used for decades to curb the development of juvenile stages (WHO 2013). LSM, which may include habitat modification and larviciding, was one of the key strategies used by countries that succeeded in eliminating malaria during the global malaria eradication campaigns in the mid-twentieth century (Killeen et al. 2013). While LSM is often perceived as resource-intensive, it can be highly effective when strategically implemented. Targeted LSM, focusing on aquatic habitats that are manageable such as stagnant and small water bodies, may be a cost-effective way of controlling juvenile water stages of mosquitoes (Fillinger et al. 2008; Fillinger and Lindsay 2011; Worrall and Fillinger 2011). On the other hand, the application of larvicides in large or fast-flowing water bodies is considered impractical due to resource constraints or the likelihood that larvicides could be washed away (Fillinger and Lindsay 2011). Thus, to enhance cost-effectiveness and precision, integration of innovative tools should be employed to help identification of high-risk larval habitats and support data-driven LSM implementation. This could ensure control efforts are focused on contexts where they are likely to yield impact. This may increase LSM efficacy and optimization as a complementary intervention, mainly in contexts where adult-focused control strategies are inadequate to interrupt malaria transmission.

#### Cutting edge technologies could enhance LSM efficacy

Cutting-edge technologies such as the use of geospatial techniques and use of drone technology to map mosquito habitats could revolutionize LSM as a mosquito control tool and reduce operational costs involved with mosquito habitat identification which may require more people. Previously, studies have highlighted the role of cutting-edge technologies such as drones, machine learning, and geospatial modelling in advancing malaria control efforts (Hardy et al. 2017; Hardy 2024). These tools facilitate high-resolution mapping of mosquito habitats and provide valuable insights on the role of waterbodies on the prevalence of vector-borne diseases (Hardy et al. 2017; DJI 2020; Joshi and Miller 2021; Stanton et al. 2021; Hardy 2024). By streamlining juvenile mosquito habitat identification, such technologies could potentially reduce the reliance on large-field teams during larval sampling or LSM interventions (DJI 2020). Furthermore, drones can accelerate larvicide applications in expansive breeding sites where manual application would be challenging (DJI 2020).

Despite these technological advancements, research on understanding mosquito biology remains mostly focused on adult stages, mainly indoor-biting vectors (Mukisa et al. 2024). Thus, majority of field studies on malaria vectors have focused on adult mosquito activities, which align with the operational hours of insecticide-treated nets (ITNs). This has been attributed to the creation of a significant gap in understanding vector behaviours during daytime periods, including juvenile mosquitoes, early evening and morning mosquito behaviours (Mukisa et al. 2024). Thus, knowledge about mosquito juveniles and the role of zoophilic and zoophagic vectors remains limited, especially within local communities. Therefore, the empowerment of communities with a holistic understanding of mosquito biology and life cycle is necessary for sustainable control. It should, therefore, be important if the same level of understanding that communities have around ITNs for indoor mosquito control should be extended to include the egg, larval, and pupal stages, as well as outdoor residual vectors (Kamndaya et al. 2021). Increased awareness campaigns on juvenile-stage control and outdoor mosquito control would enable communities to meaningfully contribute to interventions such as LSM, elimination of backyard breeding sites, and reducing mosquito exposure behaviours.

### Adult mosquitoes have a short lifespan

Mosquitoes, like many insects, have a short lifespan. While some mosquitoes may live up to 2 months, most mosquitoes will live for less than 1 month (Abd 2020; Nkahe et al. 2020; Cui et al. 2021). This short lifespan drives mosquitoes to promptly start host-seeking behaviour upon reaching a reproduction maturity (Graphical abstract) to find a potential blood meal that ensures successful reproduction (Abd 2020). While they typically mate only once throughout their lifetime, female mosquitoes can lay multiple egg batches, with each batch needing a separate blood meal to aid development (Abd 2020). This necessitates the need for repeated blood meals (Abd 2020), enhancing their vector competence, as each feeding episode increases the chance of parasite transmission. Despite their short lifespan, mosquitoes’ swift growth and repeated blood-feeding make them remarkably efficient vectors as they rapidly initiate and complete the reproductive cycle and maximize their vectorial capacity within a limited timeframe. This biological drive, consequently, presents a prevention challenge, as mosquitoes act quickly to fulfill their life cycle (National Academic Press 1991; Mitchell and Catteruccia 2017), making targeted and timely interventions critical in the control of malaria.

#### Exploiting mosquito short lifespan

Mosquitoes' swiftness could swiftness could be exploited strategically. The use of integrated vector control tools designed to disrupt mosquito-host contact can limit their access to blood meals (Musoke et al. 2018). Effective implementation of existing interventions, such as ITNs, topical repellents, and insecticide-treated clothing (Graphical abstract) can potentially reduce the frequency of mosquito bites (WHO 2019). Thus, these measures could lower the risks of malaria transmission. When widely adopted and accessible at the local community level, such interventions could offer both individual and community-wide protection, with the potential to substantially reduce malaria incidence over time (WHO 2015; Musoke et al. 2018). Thus, a multifaceted malaria control approach that involves combining various interventions and active community involvement may increase possibilities of achieving significant reductions of malaria transmission and mosquito density.

### Blood-feeding is only done by female mosquitoes

Male mosquitoes, including male *Anopheles*, do not bite as they rely exclusively on sugar and water (Foster 1995). Female mosquitoes, on the other hand, need blood in addition to sugar, as blood contains important proteins that help the maturation of their eggs (Abd 2020; Weng and Shiao 2020). Thus, without blood, mosquitoes will not reproduce (Foster 1995). This biting behaviour presents us with opportunities for mosquito control. For example, ITN use and other mosquito-bite-protective strategies like body repellents exploit this behaviour by minimizing the number of mosquitoes that have access to blood, eventually reducing mosquito density as their reproduction is reduced. The mosquito’s need for blood has been exploited for decades using ITNs and indoor residual spraying (IRS) which are currently the primary mosquito control tools. The achievements from these interventions cannot be ignored from the successes that have been accomplished over the years (Bhatt et al. 2015).

#### Residual transmission threatens routine interventions

Despite their success, the effectiveness of ITNs and IRS to drive malaria transmission towards zero is limited by the diverse and complex behaviours such as outdoor biting and change in biting patterns that allow mosquitoes to evade contact with these lethal interventions (Killeen et al. 2013; Killeen 2014a). To achieve malaria eradication, it requires that available intervention options should be able to fully interrupt the transmission of pathogens (Feachem and Sabot 2008). One way to do this is the use of complementary tools targeting both indoor and outdoor malaria vectors through integrated vector management (IVM) (Govella and Ferguson 2012; Musoke et al. 2018; Sherrard-smith et al. 2019). Although primary vector control machinery such as ITNs and IRS can suppress transmission and alleviate disease burden (Sharp et al. 2007; Killeen et al. 2007), ITNs and IRS alone are inadequate to contribute to malaria elimination in endemic settings such as in Africa where the entomological inoculation rate (EIR) can reach up to one thousand infectious bites per person per year (Smith et al. 2005, 2007). Modelling has predicted that even a 100% coverage of ITNs in an entire population may not be able to take EIR below the elimination threshold within a local community (Samir et al. 2015; Schmit et al. 2024). This sustains malaria transmission.

Malaria transmission that persists despite the achievement of universal coverage of ITNs and IRS is known as *residual transmission (*Hii et al. 2021*)*. Residual transmission is recognized as a barrier to achieve control and elimination of malaria (WHO 2023). This is also one of the biggest malaria control challenges faced by malaria control stakeholders and national malaria control programmes (NMCPs) in Africa, where a significant proportion of malaria transmission is due to residual transmission (Killeen et al. 2017a). In the recent years, we have seen an estimated 10% increase in mosquitoes that are biting outdoors, avoiding contact with the routine malaria control tools that are biassed towards indoor mosquitoes (Sherrard-smith et al. 2019). This has consequently increased yearly malaria cases by approximately 10.6 million (Sherrard-smith et al. 2019). More innovations and studies exploiting mosquito behaviours, including outdoor feeding behaviour, are required if we are to realize malaria elimination.

There is growing evidence that outdoor and residual transmission of malaria continues to undermine the effectiveness of core malaria interventions. Yet, research and the implementation of outdoor malaria control strategies remain limited in many African settings. As a result, routine tools often remain the main, and sometimes over-relied-upon tool for malaria prevention, even in contexts where their impact is reduced due to changing mosquito ecology and behaviour (Sougoufara et al. 2014).

#### Understanding local vector species is key for implementing proper control strategies

Malaria control initiatives need to be tailored to specific mosquito behaviours and ecological contexts for effective and sustainable control (Godfray 2013; Nzioki et al. 2023). Implementation of strategies without a thorough and up-to-date understanding of such factors can result in suboptimal outcomes (Godfray 2013). In some settings, interventions have been deployed without proper understanding of the local vector species, breeding habitats, ecology, biting patterns, and/or resting habits (Mapua et al. 2021a; Nzioki et al. 2023). This blanket approach does not only limit the effectiveness of control tools but also delays the implementation of more appropriate, context-specific strategies, highlighting the need for locally focused entomological surveillance to provide updated data on mosquito ecology and behaviours (Mapua et al. 2021a; Nzioki et al. 2023). Thus, national malaria programmes can better implement targeted vector control interventions tailored to the local contexts to maximize impact and closing gaps left by conventional methods.

To address this, there is an urgent need for greater engagement with national policy makers, implementation partners, and NMCPs on the importance of integrating strategies that complement routine tools. While routine tools have been instrumental in reducing the burden of malaria, continued reliance on such tools alone is no longer sufficient (Ferguson et al. 2010b; Killeen 2014a). It is critical that residual transmission is not only acknowledged but proactively addressed through integrating complementary novel strategies into national malaria control toolkits (WHO 2020). These interventions should be tailored to local ecological and behavioural context (WHO 2020). By increasing the scope of control strategies and embracing innovative solutions to tackle residual transmission, NMCPs can strengthen their efforts, moving closer towards the goal of eliminating malaria.

#### Innovative control strategies could complement conventional tools

Cutting-edge tools and innovations have been proposed to target malaria vectors that have adapted to feeding outdoors and early in the evening and morning, when people are not protected by routine interventions. Such interventions include attractive targeted sugar baits (ATSBs) (Zembere 2024) (Graphical abstract). Such tools could complement the current tools that are biassed towards indoor mosquitoes. Despite showing promise in some settings, however, ATSB performance has not been consistent, showing increased efficacy in other settings, i.e. in Mali, and reduced efficacy in other settings such as Kenya and Zambia (Müller et al. 2010a; Ogwang et al. 2025). This could potentially be due to variations in mosquito behaviour and ecology which may influence sugar-feeding preferences (Manda et al. 2007; Nyasembe et al. 2018). Additionally, variations in the availability of natural sugar sources within a location may influence how different mosquitoes behave towards ATSBs as some plants could be competitive to ATSB stations. However, these factors remain inadequately understood due to limited research in Africa (mostly Kenya and Mali (Müller et al. 2010a; Omondi et al. 2022)) to explore mosquito natural sugar feeding preferences.

To contribute towards closing this knowledge gap, more studies need to explore the suitability of ATSBs in different ecological settings with diverse natural sugar sources. A recent study (to be published separately) evaluated the sugar-feeding behaviour of *anopheline* mosquitoes in a sugarcane-growing area in southern Malawi. The study will provide valuable evidence that could inform the development and optimization of sugar-based vector control tools tailored for malaria-endemic settings such as Malawi. Among key findings of the study was that *A. gambiae* s.1. exhibits a strong attraction to *Psidium guajava* (guava) and *Musa *spp. (banana), while *Sclerocarya birrea* (marula) and *Saccharum officinarum* (sugarcane) were less attractive. These results are partially consistent with findings from a study in Mali, where *Psidium guajava* was also identified as a highly attractive sugar source for *A. gambiae* s.l. (Müller et al. 2010a). Conversely, notable differences were observed where sugarcane was one of the more attractive options in Mali (Müller et al. 2010a), contrasting with its lower attractiveness in the Malawian context. Similarly, *Musa *spp., which was less attractive in Mali (Müller et al. 2010a), was among the most attractive in Malawi. This highlights the need to understand local mosquitoes in relation to local sugar sources and could facilitate implementation of sugar-based control tools. This ensures such tools are tailored to the local ecological context to maximize efficacy. Our study will add to the available but limited evidence on mosquito sugar-feeding preferences (which is currently under studied). 

Variability in the attractiveness of local plant sugars to *Anopheles* mosquitoes (Paré et al. 2024) underscores the importance of understanding local mosquito feeding behaviours to optimize control tools. Further studies are thus crucial to better understand these dynamics across diverse settings in Africa. Such insights could explain the variations observed in the performance of ATSBs across different countries. 

Additionally, despite the continued reliance on ITNs as a frontline tool for preventing malaria, universal ITN distribution has been attributed to increased insecticide resistance due to over-reliance on pyrethroid insecticides (Oxborough et al. 2024). Universal coverage with ITNs, where majority of households have ITNs access, may overlook critical factors such as insecticide resistance profiles and outdoor biting which might undermine ITNs’ long-term effectiveness (Silalahi et al. 2022; Oxborough et al. 2024). As discussed earlier, distribution of ITNs is often done with limited consideration of the local vector ecology or insecticide resistance challenges (WHO 2019; Oxborough et al. 2024). This approach may undermine the long-term sustainability of vector control programmes. Thus, there is a pressing need to move beyond coverage statistics and invest in innovations that are tailored to enhance the effectiveness of existing tools to the local contexts (Oxborough et al. 2024). 

For example, while ITNs remain essential, their effectiveness can be improved. Innovations to improve ITNs could be explored to minimize the development of insecticide resistance in primary vectors. One potential way could be to explore the use of insecticides with different modes of action (WHO 2012a; Silalahi et al. 2022). To illustrate, a pyrethroid could be mixed with a carbamate or an organophosphate or used through rotations as part of insecticide resistance management to reduce cross resistance. However, it is important to make sure that successive mosquito generations are not exposed to insecticides with the same mode of action during rotations (IRAC 2025).

Importantly, such innovations should not be implemented in isolation. As discussed already, their success depends on up-to-date vector surveillance that can inform where and when specific insecticide combinations or strategies are appropriate. This could be based on local resistance profiles and mosquito behaviour (Burkot et al. 2019; Hancock et al. 2024). By coupling resistance management to routine vector surveillance and localized ecological knowledge, malaria programmes can shift from reactive to proactive control, an approach currently lacking in most malaria-endemic regions (Dusfour et al. 2019).

Another vector control method that can exploit the feeding behaviour of mosquitoes is the use of endectocides (Graphical abstract) which are antiparasitic drugs that are active against parasites such as the malaria parasite, *Plasmodium *sp. (Foy et al. 2011). When given to humans or livestock as part of mass drug administration for the control of nematodes, endectocides have additional benefits, rendering human or animal blood meals toxic for the mosquito vector (Foy et al. 2011). In recent years, there has been an increase in studies interested in exploring the potential of using endectocides such as ivermectin for malaria control in livestock such as cattle (Chaccour and Killeen 2016; Njoroge et al. 2017; Ruiz-Castillo et al. 2022). Administering ivermectin to animals is a potential One Health approach as it may help improve the health of domestic animals while indirectly improving human health by controlling parasites such as *Plasmodium falciparum* (Ruiz-Castillo et al. 2022). Endectocides can kill the mosquito, or negatively affect mosquito fitness, fertility, and can also inhibit development of the *P. falciparum* in the mosquito (Slater et al. 2020).

While IRS has effectively targeted indoor resting behaviour of mosquitoes, outdoor resting behaviour remains understudied, presenting a significant gap in current vector control tools. This is becoming more challenging with the increasing evidence that many malaria vectors are becoming adapted to biting and resting outdoors, reducing the effectiveness of ITNs and IRS (Russell et al. 2011; Killeen 2014b; Killeen et al. 2017b).

While there is an increase in the number of studies that have concentrated on surveillance of mosquito behaviour and resting preferences in outdoor settings in Africa (Degefa et al. 2019; Mapua et al. 2021b), notably, there is still a lack of practical and field-based interventions particularly targeting outdoor mosquito resting sites such as peri-domestic structures and livestock shelters. This highlights the need for applied interventional studies focusing on outdoor-based vector control strategies to complement current indoor-based tools like ITNs and IRS. Additionally, most of these studies have been limited to specific settings, requiring an expansion of research in this area to unlock new opportunities for integrated vector control that suit specific local contexts. When such interventions are coupled with other outdoor based initiatives such as the use of endectocides (Chaccour and Killeen 2016; Ruiz-Castillo et al. 2022), they could enhance the impact of malaria control programmes by addressing mosquito vectors that evade conventional strategies.

### Mosquitoes can take blood up to three times their body weight

One of the fascinating characteristics of the mosquitoes is the ability to take up blood volumes up to three times their body weight during a single feeding session. This is facilitated by the expansion of their stomachs which expand when feeding on blood (Gonzales et al. 2018). Putting this into perspective, it would be equivalent to a 50 kg human being consuming food weighing 150 kg in a single sitting. Thus, if the previous blood-feeding attempts were not successful, this substantial blood requirement often necessitates multiple blood-seeking attempts. This increases the likelihood of mosquitoes biting several individuals. Recent genotyping and microscopy studies on blood meal analysis of malaria mosquitoes in Malawi and Malaysia, respectively, have shown that mosquitoes can take several blood meals if previous attempts were not successful (Jeyaprakasam et al. 2022; Mbewe et al. 2023). Therefore, the more homes are protected by ITNs and IRS, the higher the chances that mosquitoes visiting such homes, seeking blood meals, are likely to be exposed to and thus killed by chemicals in ITNs and IRS. Although IRS is expensive to use in highly malaria-endemic settings such as the typical African communities, ITNs are relatively cheap and easily accessible (WHO 2022a). In most African countries, ITNs are distributed to local communities approximately every 3 years as part of routine malaria control campaigns (Koenker et al. 2023).

As ITNs are the primary malaria control tools, the global malaria fight has been principally geared towards using them (Onyinyechi et al. 2023). ITN use has proven as the most cost-effective malaria prevention method (Nyarango et al. 2006; WHO 2014) making it the most supported malaria vector control intervention in malaria-endemic countries (WHO 2012b, 2014). As already discussed, ITN use should still be supported but should not only be the main vector control tool in contexts where the vectors are primarily endophagic. It should be complemented with other outdoor-based interventions (Govella et al. 2010; Govella and Ferguson 2012; Kaindoa et al. 2021).

 However, the use of ITNs has generally plateaued since 2016 in Africa (Bertozzi-Villa et al. 2021). Some of the factors contributing to low ITN usage could vary from socio-economic reasons to behavioural characteristics that make people avoid ITNs or use them for other purposes other than mosquito control. Thus, lack of ITN access, warm temperatures (associated with most malaria-endemic settings), poor perception of ITNs (i.e., claiming that ITNs reduce fertility rates of couples), and lack of basic malaria control knowledge are some of the factors that have been reported previously as barriers to ITN use in some parts of Africa (Slater et al. 2020; Nkahe et al. 2020; Cui et al. 2021). Another study has previously reported behavioural factors involving misuse of ITNs as people use ITNs as substitutes for curtains and fishing nets or sell them after receiving from distribution campaigns (Killeen et al. 2013).

### Adult mosquito age matters

Knowledge of the age structure of mosquito populations is important for control of malaria. Only old mosquitoes have the capability to transmit diseases (Johnson et al. 2020). This is because a mosquito is not born with parasites*.* They get parasites once they bite an infected individual. As already highlighted above, mosquitoes take their first blood meal for the development of eggs (Abd 2020), which can happen mostly during their 4th or 5th day after emergence from water. Once they get infected with the parasites, the parasites cannot be directly transmitted to another person as they will need to undergo several development stages until they reach the infective stages, a process that can take up to 10 days (Johnson et al. 2020). This means a young mosquito is less likely to transmit the malaria parasites. Therefore, if proper prevention strategies that target young mosquitoes are put in place, few mosquitoes would manage to grow old enough to transmit; thus, control of malaria can be achieved. Implementation of the novel ATSBs which are more likely to kill sugar-seeking young mosquitoes (Graphical abstract) could potentially help minimize mosquito density by reducing the population of mosquitoes that grow old enough to transmit diseases (Müller et al. 2010c). If infected malaria mosquitoes are killed before the parasites inside them reach the sporozoite stage, then malaria transmission would be reduced, contributing to malaria control.

## Other recommendations and conclusions

In line with the theme for 2025 World Malaria Day, “Malaria Ends With Us: Reinvest, Reimagine, Reignite”, there is an urgent need for renewed commitment and innovations focused on novel and context-specific control tools. This global call emphasizes the importance of revitalizing efforts at all levels, beginning from international policy making, national policy making to the grassroot level. Thus, we need to look back over the years and see where we are coming from and where we are going in as far as control of malaria is concerned. The battle against malaria mosquitoes requires joint hands by researchers, funders, governments, communities, and all stakeholders to put more resources, innovations, and energy in the fight against mosquitoes.

In line with the them of the 2023 World Malaria Day, “Time to Deliver Zero Malaria: Invest, Innovate, Implement,” there is a great need for robust investments in new tools that will facilitate the malaria elimination goal. Innovative approaches such as the use of the ATSBs (Attractive Targeted Sugar Bait Phase III Trial Group 2022) could revolutionize malaria control. Preliminary results on ATSBs have highlighted a great potential both in the laboratory and field, with some studies reporting over 80% mosquito reduction and over 50% reduction in malaria incidence (Müller et al. 2010b; Stewart et al. 2013). It is expected that the ATSB might achieve the WHO-prequalification listing (WHO 2022a), as one of the complementary malaria control tools to target residual mosquitoes not targeted by routine tools.

Additionally, applying para-transgenesis techniques using novel methods like CRISPR/Cas9 (Sander and Joung 2014) could be exploited to change the mosquito microbiome as a potential strategy for vector control. This could be achieved by knocking out microbial genetic material responsible for vectorial capacity in vectors. Transfecting malaria mosquitoes with transgenic bacteria such as *Wolbachia* could also potentially be explored for malaria control. Learning from the success of *Wolbachia* in dengue control (Iturbe-Ormaetxe et al. 2011), there is a great need for more experiments to determine if this could have an application in malaria mosquitoes as well although previous trials in *Anopheles* were unsuccessful due to limitations of *Wolbachia*’s ability to induce cytoplasmic incompatibility (CI) in *Anopheles* (Gomes et al. 2017). This challenges the release of *Wolbachia*-infected *Anopheles* mosquitoes, requiring further research to develop genetically modified *Wolbachia* that can naturally induce CI in *Anopheles* mosquitoes (Gomes et al. 2017).

With the recent invasion of *Anopheles stephensi* in Africa (WHO 2022b), malaria elimination goals seem threatened. The threat is partly due to their adaptation to breeding in urban environments, including breeding in unused tyres (Graphical abstract). Due to an increase in unplanned cities across Africa, the threat posed by this species to Africa is palpable (WHO 2022b; Mosquito matters 2022; Ochomo et al. 2023). As such, there is a need for African governments, researchers, and everyone including those in countries that have not yet reported the presence of this species to sound the alarm and put up measures to contain it.

This article recommends integrated vector management (IVM) approaches as one control strategy might not be effective. IVM approaches involve making rational decisions that optimize resource use in vector control implementation (WHO 2012c). It aims at contributing to achievement of the global malaria control targets by using local evidence and easily available resources, making vector control more efficient, cost-effective, and sustainable (Beier et al. 2008; WHO 2012c). Integrated control measures for malaria vectors could include approaches that target all life stages of the mosquitoes (Graphical abstract). They could involve LSM to target mosquitoes from their juvenile stages (to minimize the population that make it to adult stages), coupled with approaches such as the ATSBs that target young and newly emerged sugar seeking mosquitoes and strategies targeting old blood-seeking mosquitoes both indoors and outdoors such as ITNs, IRS, and mosquito repellents (Shiff 2002; Beier et al. 2008; WHO 2012c). Together, these could potentially reduce the density of mosquitoes that survive to complete the extrinsic incubation period, minimizing both mosquito density and parasite transmission (Müller et al. 2010c; Beier et al. 2012).

### Insecticide resistance and the future of vector control: gaps and priorities

Insecticide resistance is a big threat to the success of ITNs and IRS, both of which depend on a limited set of insecticide classes (WHO 2022c). It arises following repeated exposure of vectors to insecticides, allowing individual vectors with resistance traits to endure and survive and consequently reproduce. Over time, the resistant populations become dominant, reducing the impact of control interventions.

There are multiple mechanisms through which resistance can develop. These may include target-site mutations, such as behavioural changes (limiting contact with treated surfaces), knock down mutations (*kdr* mutations), and metabolic resistance (WHO 2022c). These compromise the efficacy of insecticides and lead to vector species composition shifts or changes in vector behaviour, which further complicates vector control efforts.

Management of insecticide resistance is critical but difficult. This requires coordinated approaches such as rotation of insecticides with different modes of action in IRS, deployment of combination products (such as dual-treated ITNs), and integration of other tools such as LSM and house improvement (WHO 2017a). Nevertheless, the success of such approaches depends on robust national capacity for routine monitoring of resistance, access to alternative combination products, and the proper use of surveillance data for decision making guidance (WHO 2017a, 2022c).

Despite guidance from the world health organization (WHO) and frameworks such as the global plan for insecticide resistance management (GPIRM), many low-income countries (which are also malaria-endemic) have challenges in implementing resistance management due to limited entomological capacity, resources, and poor infrastructure (WHO 2017a).

To sustain the efficacy of existing tools and ensure long-term impact, there is need that national programmes should prioritize insecticide resistance monitoring, build capacity for entomological surveillance and response, and invest in new insecticides and delivery methods (WHO 2017a).

### Mosquito surveillance: a critical driver of vector control

Entomological surveillance may provide an up-to-date information of key entomological indicators. The indicators include such as mosquito abundance (i.e., number of mosquitoes in a population), vector species composition (i.e., *A. funestus*, *A. arabiensis*, *A. gambiae* s.s.), human biting rate (HBR) (how often mosquitoes feed on humans), sporozoite rate (the proportion of mosquitoes infected with malaria parasites), entomological inoculation rate (EIR) (HBR × sporozoite rate), and insecticide resistance profiles (which is crucial for choosing suitable vector control tools) (MEI 2020; Kouassi et al. 2023). Surveillance of adult mosquitoes is thus critical for guiding vector control strategies, by informing decision making on which control tools to deploy, when and where to deploy them (MEI 2020; Kouassi et al. 2023). Thus, surveillance data can help in determining whether ITNs or IRS are suitable and where these should be prioritized. Additionally, this knowledge can guide whether there is need for complementary strategies such as LSM or spatial repellents in response to increased outdoor-biting vectors, underscoring the role of mosquito surveillance in enabling targeted and context-specific malaria control (MEI 2020; Kouassi et al. 2023).

Despite this, surveillance of adult mosquitoes is often underutilized particularly in endemic countries due in part to funding gaps and limited entomological capacity (Coulibaly et al. 2023). Nevertheless, it is critical to invest in robust entomological surveillance systems and integrate these into NMCPs if we are to ensure that interventions are timely and locally suitable and to enhance their impact and sustainability (MEI 2020; Kouassi et al. 2023). The World Health Organization Global Technical Strategy for Malaria (2021–2030) and the Global Vector Control Response (GVCR) 2017–2030 recommend surveillance systems incorporating entomological and epidemiological surveillance, advocating that surveillance should become a core intervention (WHO 2017b, 2021).

### Other complementary outdoor vector control tools: enhancing IVM

While indoor-based tools like IRS and ITNs remain fundamental for malaria control (Bhatt et al. 2015), effective IVM strategies require complementary strategies tailored at targeting mosquitoes in different ecological settings and at multiple life stages (WHO 2004, 2011, 2012c). Additional tools such as ultra-low volume adultcides spraying (ULV) and LSM using larvicides could play important roles in targeting mosquitoes outside the reach of routine interventions (Fillinger and Lindsay 2011; Preftakes et al. 2011; Tusting et al. 2013).

Larviciding using *Bacillus thuringiensis israelensis* (Bti) has shown success by reducing adult mosquito emergence in several settings. Bti, a biological method, is cost-effective, species-specific, and environmentally safe, increasing its operational acceptance by communities (Mpofu et al. 2016; Derua et al. 2019; Brühl et al. 2020). Additionally, larviciding agents such as temephos (Marina et al. 2014) are also effective though their operational use is constrained in part by cost and frequency of application, particularly in low-income settings, due to limited donor support.

Despite inadequate donor support, larviciding remains a vital tool for IVM, particularly in urban and peri-urban areas where larval habitats are accessible and well-defined (Castro et al. 2010). Furthermore, LSM is most effective when complimented with surveillance and community-based reporting of larval habitats (WHO 2013).

Furthermore, novel outdoor interventions such as ultra-low volume (ULV), which involve spraying small quantities of insecticides, dispersing fine droplets that target adult mosquitoes in outdoor settings, in addition to ATSBs, ivermectin, and other proposed outdoor tools could complement routine tools like ITNs and IRS (Shiff 2002; WHO 2004; Beier et al. 2008; Preftakes et al. 2011; WHO 2012c). While the efficacy of ULV against malaria vectors is debatable, its use in dengue and other arboviral diseases has been successful (Farajollahi et al. 2012). However, deploying ULV during periods of peak mosquito activity and following the right formulation have shown efficacy in reducing outdoor malaria vector densities (Chaskopoulou et al. 2011).

### Community engagement is key

Community engagement is critical for sustainable malaria control. Empowering community members with basic knowledge of mosquito biology and ecology, and how these relate to disease transmission, is essential in enhancing community-driven prevention efforts (Godfray 2013; Nganga et al. 2019; Awasthi et al. 2024). A good example is the human animator approach, where local volunteers were trained to identify malaria mosquitoes and larval habitats in Malawi (Malenga et al. 2017; Kaunda-Khangamwa et al. 2019; Tizifa et al. 2022). Whereas high-tech tools such as remote sensing, machine learning, and drones could potentially optimize LSM (Hardy et al. 2017; Stanton et al. 2021; Hardy 2024), simple and low-tech tools such as dippers and locally made scoops remain effective and should not be overlooked (WHO 2013). These could remain critical components when embracing community-led vector surveillance and control. Such tools are affordable and easy to use and can empower local communities to identify and monitor larval habitats (WHO 2013). This may not only promote early detection but also support timely and targeted interventions and strengthen grassroot participation in malaria control, fostering ownership of control initiatives (WHO 2013).

This model can be expanded to include training community members on how to identify both primary and secondary malaria vectors and understanding mosquito behavioural shifts, such as increased zoophilic and zoophagic behaviours, which reduces the protective efficacy of ITNs.

A community equipped with this knowledge can make more informed decisions such as avoiding late-night exposures or outdoor sleeping and supporting the adoption of complementary outdoor vector control measures (Monroe et al. 2015). Therefore, malaria-related health promotion and education interventions must go beyond encouragement of ITN use to encompassing broader topics like behaviour change, vector ecology, and environmental management (Monroe et al. 2015; Abrar Hamza 2017; Ingabire et al. 2017). Thus, NMCPs and government health departments should prioritize culturally tailored health education strategies. This is critical not only to improve the correct usage of ITNs, but also to build long-term community resilience and ownership of malaria vector control efforts, which is crucial as we strive to eliminate malaria (Ingabire et al. 2017).

### Policy recommendations: capacity building

One of the barriers for malaria elimination and control of vector-borne diseases in Africa is the limited number of trained local vector biologists (Killeen et al. 2002; George and Bockarie 2022). This creates a gap and limits the capacity of national programmes to conduct context-specific surveillance and control initiatives. Additionally, this limits the ability to understand vector-specific behaviours and tailoring of control strategies to meet local ecological needs, resulting in implementation of intervention without addressing the ecological and behavioural diversity of vectors (Killeen et al. 2002; George and Bockarie 2022).

Therefore, community engagement needs to extend beyond awareness and participation of local community members but should include local scientific empowerment (Killeen et al. 2002). Investing in capacity building to train new vector biologists, particularly at postgraduate level, could bridge this gap. Thus, there is a need to facilitate training in research and involvement in capacity building on issues that link fundamental insect biology with human diseases in low-income countries. For example, PhD trainees could focus their research on the development and field testing of novel surveillance and monitoring tools targeting both indoor and outdoor vectors, and the application of innovative vector control technologies.

This may help to strengthen local expertise in Africa, equipped to generate context-specific scientific evidence, guide proper vector control strategies, and reduce reliance on approaches that are externally driven (Killeen et al. 2002; George and Bockarie 2022). In the long term, this skilled workforce of vector biologists would not only improve the vector control work force but also boost regional research capacity, innovation, and mentoring of future scientists. This could ensure sustainability and independence in vector-borne disease management, critical for vector-borne disease elimination. However, stable funding is also essential to retain expertise and support entomological infrastructure, ensuring continuity of vector control programmes and mosquito surveillance (WHO 2017b). Unreliable financial support may lead to reduced impact of interventions even when there are well-trained vector biologists and innovative ideas. Capacity building should therefore go hand in hand with stable funding as this is essential for resilient vector control efforts (WHO 2017b).

## Data Availability

No datasets were generated or analysed during the current study.
